# The association of physical activity, sedentary behaviors, and body mass index classification in a cross-sectional analysis: are the effects homogenous?

**DOI:** 10.1186/1471-2458-11-926

**Published:** 2011-12-14

**Authors:** Justin B Dickerson, Matthew Lee Smith, Mark E Benden, Marcia G Ory

**Affiliations:** 1 Department of Health Policy & Management, School of Rural Public Health, Texas A&M Health Science Center, College Station, Texas, USA; 2 Department of Health Promotion & Behavior, College of Public Health, The University of Georgia, Athens, Georgia, USA; 3 Department of Environmental & Occupational Health, School of Rural Public Health, Texas A&M Health Science Center, College Station, Texas, USA; 4 Department of Social & Behavioral Health, School of Rural Public Health, Texas A&M Health Science Center, College Station, Texas, USA

## Abstract

**Background:**

While much is known about the benefits of physical activity (PA) and the consequence of sedentary behaviors relative to body mass index (BMI), little is known about the homogeneity of these effects across individuals. The goal of this study was to determine if PA and sedentary behaviors have the same effect on individuals of all BMI classifications.

**Methods:**

Data from a community health assessment were analyzed and a sample was selected to include respondents who self-reported a chronic disease associated with obesity (n = 2,840). Descriptive statistics were used to describe the association between selected independent variables and BMI. Simultaneous quantile regression was used to identify the degree of homogeneity in the effect of demographic independent variables, minutes per week of moderate PA, and hours per day spent watching television on BMI classification. In studies using simultaneous quantile regression, the word "effect" is used to describe association, not causation.

**Results:**

Minutes per week of moderate PA had a significant effect on lower BMI, but only when respondents were at least classified as obese-class I (β = -0.001, *p *= 0.006). The change in effect of moderate PA in lower BMI increased significantly when respondents were classified as obese-class II versus obese-class I (F = 4.54, *p *= 0.033). Hours per day spent watching television had a significant effect on higher BMI, but only when the respondent was at least classified as overweight (β = 0.87, *p *< 0.001). The change in effect of watching television on higher BMI increased significantly when respondents were classified as obese-class I versus overweight (F = 5.57, *p *= 0.018).

**Conclusion:**

PA and watching television were more related to BMI for obese individuals than those who were just overweight. Customized interventions for specific BMI classifications should be developed to maximize public health benefits.

## Background

The last decade witnessed a noteworthy increase in body mass index (BMI) with average age-adjusted BMI for American adults now reported as 28.5 kg/m^2 ^[1]. This is alarming considering the World Health Organization (WHO) recognizes a BMI of 30.0 kg/m^2 ^as obese [2]. The effects of obesity on mortality are substantial. Not only is obesity a risk factor for cardiovascular disease [3], it has been shown that more time spent as an obese person is directly related with increased risk of all-cause mortality [4]. As a result, obesity places an economic strain on the healthcare system and society as a whole. It is estimated that total economic effects of obesity on the American economy are more than $200 billion annually [5].

The WHO uses BMI to classify individuals as either underweight (BMI < 18.5 kg/m^2^), normal weight (BMI of 18.5-24.99 kg/m^2^), overweight (BMI of 25.0-29.99 kg/m^2^), obese class I (BMI of 30.0-34.99 kg/m^2^), obese class II (BMI of 35.0-39.99 kg/m^2^), or obese class III (BMI >= 40.0 kg/m^2^) [2]. While obesity has increased substantially over the past decade, the increase in obese class III, also known as "morbid obesity," has increased at twice the rate of lower obese classifications [6]. Research indicates morbidly obese individuals suffer from serious co-morbidities with nearly half (48%) diagnosed with hypertension, 29% diagnosed with diabetes, and 25% diagnosed with heart failure [7]. Treating many of these conditions is difficult because morbidly obese individuals are often physically unable to avail themselves of modern diagnostic medicine because of their size, especially when attempting to be treated for cardiovascular diseases [8]. It is therefore understandable that mortality rates of hospitalized morbidly obese individuals are increasing and are highest among those with the highest BMI [7]. These findings provide context to research indicating healthcare expenditures for the morbidly obese are 47% greater than for the obese class I population [9].

There are many causes for increasing rates of obesity. Among these risk factors are sedentary behaviors [10]. Even though engaging in positive physical activity (PA) habits such as exercise, individuals who simultaneously engage in sedentary behaviors (e.g., such as extended hours watching television or sitting in an office or car) can still experience negative health consequences [11]. Research indicates only 3% of waking hours are currently spent on exercise while 58% are spent engaging in sedentary behaviors such as sitting or lying down [10]. Based on these findings, researchers have argued sedentary behaviors should be considered an independent set of risk factors [12] related to both all-cause and cardiovascular disease mortality [13]. Among sedentary behaviors, extended periods watching television has been uniquely linked to increasing rates of obesity [14]. Even when adjusted for socio-demographics, co-morbidities, and family history, each 1 h per day increase in time spent watching television has been positively associated with increased risk of all-cause mortality and cardiovascular mortality [15].

It is important to recognize the role PA (e.g., exercise) can play in mitigating negative health outcomes. PA has been linked with lower risk of heart disease and certain cancers, and has also been identified as an effective tool in addressing obesity [16]. The current federal guidelines for PA recommend 150 min per week of moderate intensity activity or 75 min per week of vigorous intensity activity [17]. Despite these recommendations, however, there is an inverse relationship between BMI and the likelihood of exercising [18]. This represents a key challenge for healthcare providers to address.

The research identified above is demonstrative of growing literature linking sedentary behaviors, physical inactivity, and obesity. However, what is less emphasized in the literature is the relationship of these variables to individuals of differing levels of obesity. The purpose of our study is to examine whether there is a homogenous effect between sedentary behaviors, PA, socio-demographic variables and BMI classification. This research question is important to policymakers as it may assist in targeting interventions to certain BMI classifications, and in return maximize the use of financial resources to address obesity.

## Methods

### Brazos valley health assessment (BVHA)

The 2010 BVHA (n = 3,964) was conducted and funded by the Center for Community Health Development at the Texas A&M Health Science Center, School of Rural Public Health [19]. In conjunction with community health partners, a voluntary questionnaire was disseminated to assess community health status and opportunities for health improvement in the Brazos Valley, an eight county region in central Texas [19]. Data were collected using a random sampling of households. The recruitment specialist solicited the head of household according to the adult with the next coming birthday in order to eliminate bias. Based on the head of household's eligibility and consent to participate, a survey instrument was mailed to them. Only one survey instrument per household was collected. The instrument was 32 pages containing items from validated sources such as the Center for Disease Control and Prevention's National Health and Nutrition Examination Survey (NHANES) and Behavioral Risk Factor Surveillance System (BRFSS) [19-21].

### Sample

Respondents who self-reported being diagnosed by their healthcare provider with a disease or medical condition associated with obesity were included in our study (n = 2,840). These diseases or medical conditions were: hypertension, heart failure, high cholesterol, angina, heart attack, stroke, diabetes, asthma, and chronic obstructive pulmonary disorder. Respondents who self-reported severe impairment such as loss of hearing or eyesight, a loss of limb(s), and respondents not reporting data to calculate a BMI or reporting data resulting in a BMI classification of underweight were excluded from the sample (n = 859). This exclusionary criteria was used because several of the variables in the study (e.g., watching television and PA) involved the use of physical faculties and the investigators were unaware of the degree such physical impairments would naturally impede participation in these activities, potentially resulting in biased study results.

### Dependent variable

The dependent variable was BMI classification. All sample respondents self-reported their height and weight, which were then used to calculate their BMI. They were then assigned to BMI classifications based on the methodology used by the WHO [2] (i.e., underweight (BMI < 18.5 kg/m^2^), normal weight (BMI of 18.5-24.99 kg/m^2^), overweight (BMI of 25.0-29.99 kg/m^2^), obese class I (BMI of 30.0-34.99 kg/m^2^), obese class II (BMI of 35.0-39.99 kg/m^2^), or obese class III (BMI >= 40 kg/m^2^). WHO classifications of obesity were used because of the range of classifications (i.e., six categories) versus alternative classification systems such as the one offered by the Centers for Disease Control and Prevention which only has four categories [22]. A wider range of classifications allowed for more precise estimates using our statistical model described below because it improved the within group homogeneity of subjects within each BMI classification.

### Independent variables

The independent variables were chosen based on established literature indicating an association with BMI. Age, sex, and race/ethnicity were selected as independent variables based on research identifying their importance to obesity [23]. Age was measured as a continuous variable while sex and race/ethnicity were measured as categorical variables. The categories of race/ethnicity were non-Hispanic White, African-American, and Hispanic. Educational attainment was selected as an independent variable based on research examining its role as a covariate of obesity rates [1]. Educational attainment was measured as a continuous variable from one (first grade educational attainment) to 17 (graduate school educational attainment). Rurality was selected as an independent variable based on the differing food environments of urban and rural locales and the associated relationship with rates of obesity [24]. Rurality was measured as a categorical variable by county and then matched to an Urban Influence Code [25] which designated the respondent as living in a county that was either rural or urban. Hours per day spent watching television was selected as an independent variable to be representative of sedentary behavior as part of our research question. Hours per day spent watching television was measured as a categorical variable using a six category response format (Question PAQ.710) in accordance with CDC's validated NHANES instrument [20]. The categories in the BVHA were: less than 1 h, 1-2 h, 2-4 h, 4-6 h, and more than 6 h. Finally, minutes per week of moderate PA (e.g., fast walking) was selected as an independent variable as part of our research question. Minutes per week of moderate PA was measured as a continuous variable. This question was based on the CDC's BRFSS [21] survey to enhance validity of the resulting data.

### Descriptive statistics

Chi-square, *t*-test, and Kruskal-Wallis statistics were used to examine the relationships between independent variables and the dependent variable. Statistical significance was established as α = 0.05, but was then adjusted using the Bonferroni correction. Based on seven comparisons in the descriptive statistics analysis, the new level of statistical significance was (0.05/7) = α = 0.007.

### Simultaneous quantile regression

In studies using simultaneous quantile regression, the word "effect" is used to describe association, not causation. To remain consistent with established literature, we are also using the term "effect" to imply association but not causality. The research question of the study required an estimation of homogeneity of effect between the independent variables and BMI classification. This required estimators that not only recognized effects at all BMI classifications, but estimators that could also recognize effects at specific points in the dataset. We selected simultaneous quantile regression because of its unique ability to meet our analytical requirements. Specifically, we choose simultaneous quantile regression over quantile regression because it enabled us to test for the similarity of effect of our independent variables at different points in the dataset. This is a unique facet of simultaneous quantile regression relative to quantile regression [26,27]. We used the "sqreg" command in Stata Version 11 (StataCorp, College Station, TX) for our analysis and required the model to perform 100 repetitions of the analysis to provide more accurate bootstrapped standard errors. Any respondents containing missing data in the analysis were listwise deleted from the analysis in Stata Version 11 (StataCorp, College Station, TX). Because simultaneous quantile regression estimates the effect of the independent variables at multiple points in a dataset, it was important to know which points in the dataset to assign to each BMI classification. We chose the median point of each classification. Thus, if those who were normal weight were represented between the zero and the 20th percentile in the dataset, we set the point of analysis for normal weight at the 10th percentile.

### Graphically representing effects by BMI classification

**Figure 1:**
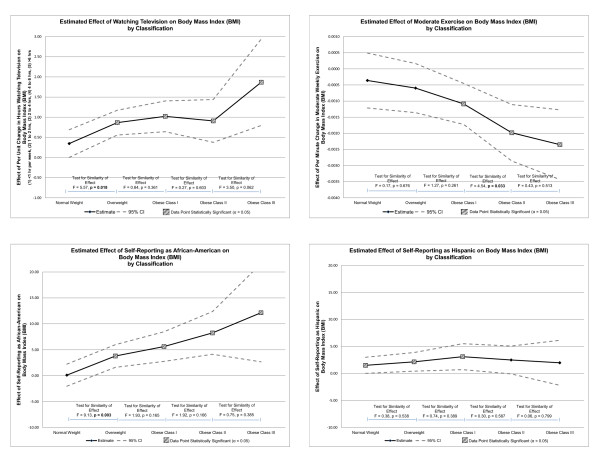
**Effect of independent variables on body mass index by classification**.

**Figure 2:**
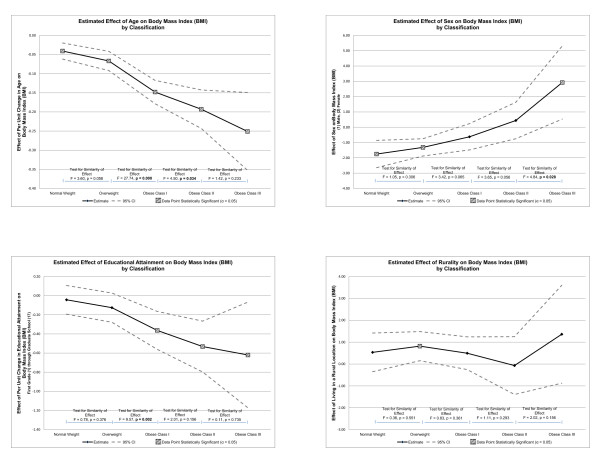
**Effect of independent variables on body mass index by classification, continued**.

The results of the simultaneous quantile regression model are represented graphically for ease of interpretation (please see Figures 1 and 2). Each independent variable coefficient and respective *p*-value was plotted as an estimate of effect on its own line graph and included a 95% confidence interval. The vertical axes of the graphs represent the effect of the independent variable on BMI, while the horizontal axes represent the BMI classification. Data points representing *p*-values of statistical significance (i.e., α = 0.05) were indicated in the legend. Finally, a series of separate calculations was performed to measure the similarity of effect between each BMI classification. An F-statistic was reported along with a *p*-value to test for similarity, or homogeneity, of effect between classifications. The null hypothesis was homogeneity of effect. Statistical significance of α = 0.05 was used to reject the null hypothesis, and indicate heterogeneity of effect between classifications. Each line graph was assimilated into two quadrant figures for ease of review (please see Figures 1 and 2).

## Results

### Sample

Employing selection criteria resulted in 1,981 respondents in the analytical sample. Table 1 reports the results of the descriptive statistics analysis. Of the respondents, 477 were classified as normal weight, 664 were classified as overweight, 427 were classified as obese class I, 177 were classified as obese class II, and 236 were classified as obese class III. The mean age of the respondents was 59.96, with the youngest respondents belonging to obese class III (54.65 ± 13.61). The majority of respondents were: female, non-Hispanic White, and more than half attended some college. Most respondents also lived in urban areas. On average, the majority of respondents exceeded federal guidelines for weekly minutes of moderate PA. However, nearly a quarter of respondents spent more than 4 h per day watching television. With the exception of rurality, each comparison made between the independent variables and BMI classification was statistically significant at the α = 0.007 level. Since the null hypothesis assumes equality between the different BMI classifications for each of the independent variables, a test statistic producing a *p*-value below the alpha value indicates the null hypothesis is rejected and each BMI classification exhibits differences to the others on each of the independent variables. This is an appropriate finding as we would expect the BMI classifications to vary on multiple variables. Using an alpha value of 0.007 reflects the traditional use of the 0.05 level of statistical significance adjusted for the multiple comparisons in the statistical test. Dividing 0.05 by the seven comparisons in the test results in a more stringent threshold of 0.007. This ensures maximum confidence when rejecting the null hypothesis.

**Table 1:** Descriptive Statistics by Body Mass Index (n = 1,981)

Body Mass Index Classification (World Health Organization)								
	**Normal Weight**	**Overweight**	**Obese Class I**	**Obese Class II**	**Obese Class III**	**Total**	**X**^**2**^	***p***
	**(n = 477)**	**(n = 664)**	**(n = 427)**	**(n = 177)**	**(n = 236)**	**(n = 1,981)**		
*Age	61.96 (±15.00)	61.70 (±13.34)	59.09 (±12.95)	56.45 (±10.88)	54.65 (±13.61)	59.96 (±13.73)	71.837	**<0.001**
Sex							43.79	**<0.001**
Male	107 (22.4%)	236 (35.5%)	151 (35.4%)	58 (32.8%)	38 (16.1%)	590 (29.8%)		
Female	370 (77.6%)	428 (64.5%)	276 (64.6%)	184 (67.2%)	174 (73.7%)	1,367 (69.0%)		
Not Provided	0 (0.0%)	0 (0.0%)	0 (0.0%)	0 (0.0%)	24 (10.2%)	24 (1.2%)		
Race/Ethnicity							124.47	**<0.001**
Non-Hispanic White	409 (85.8%)	558 (84.1%)	341 (79.9%)	136 (76.8%)	130 (55.1%)	1574 (79.4%)		
African-American	32 (6.7%)	44 (6.6%)	46 (10.8%)	18 (10.2%)	44 (18.6%)	184 (9.3%)		
Hispanic	23 (4.8%)	46 (6.9%)	33 (7.7%)	19 (10.7%)	37 (15.7%)	158 (8.0%)		
Not Provided	13 (2.7%)	16 (2.4%)	7 (1.6%)	4 (2.3%)	25 (10.6%)	65(3.3%)		
*Educational Attainment	13.68 (±2.68)	13.81 (±2.46)	13.62 (±2.50)	13.32 (±2.40)	12.34 (±2.91)	13.53 (±2.60)	43.6	**<0.001**
(1 = First Grade, 17 = Graduate School)								
Rurality							3.97	0.41
Urban	302 (63.3%)	410 (61.7%)	251 (58.8%)	102 (57.6%)	152 (64.4%)	1217 (61.4%)		
Rural	175 (36.67%)	254 (38.3%)	176 (41.2%)	75 (42.4%)	84 (35.6%)	764 (38.6%)		
Hours per day spent watching television							54.03	**<0.001**
Less than 1 h	60 (12.6%)	72 (10.8%)	30 (7.0%)	15 (8.5%)	19 (8.1%)	196 (9.9%)		
1-2 h	142 (29.8%)	198 (29.8%)	109 (25.5%)	34 (19.2%)	45 (19.1%)	528 (26.7%)		
2-4 h	165 (34.6%)	247 (37.2%)	149 (34.9%)	73 (41.2%)	62 (26.3%)	696 (35.1%)		
4-6 h	57 (11.9%)	87 (13.1%)	81 (19.0%)	29 (16.4%)	38 (16.1%)	292 (14.7%)		
More than 6 h	43 (9.0%)	49 (7.4%)	48 (11.2%)	24 (13.6%)	38 (16.1%)	202 (10.2%)		
Not Provided	10 (2.1%)	11 (1.7%)	10 (2.3%)	2 (1.1%)	34 (14.4%)	67 (3.4%)		
*Minutes per week of moderate physical activity	209.27 ± (328.32)	199.19 ± (366.12)	206.99 ± (422.37)	165.06 ± (381.67)	109.05 ± (291.52)	190.87 ± (365.95)	77.65	**<0.001**

* Chi-squared statistics based on the Kruskal-Wallis equality of populations rank test

Note--Statistical significance is determined using the Bonferroni correction. Based on 7 comparisons in the analysis, the level of statistical significance is α = 0.007

### Simultaneous quantile regression--overall model results

Table 2 reports the results of the simultaneous quantile regression model. Results are reported by BMI classification. For those of normal weight, respondents were more likely to be older, female, and self-report as Hispanic. For those classified as overweight, respondents were more likely to be older, female, self-report as non-Hispanic White, African-American or Hispanic, live in a rural location, and spend more hours per day watching television. For those classified as obese class I, respondents were more likely to be younger, self-report as non-Hispanic White, African-American or Hispanic, to have attained less education, spend more hours per day watching television, and spend fewer minutes per week engaged in moderate PA. For those classified as obese class II, respondents were more likely to be younger, self-report as African-American, to have attained less education, to spend fewer minutes per week engaged in moderate PA, and spend more hours per day watching television. Finally, for those classified as obese class III, respondents were more likely to be younger, female, self-report as African-American, to have attained less education, to spend fewer minutes per week engaged in moderate PA, and spend more hours per day watching television. These results indicate a general pattern of PA and sedentary behavior coefficients becoming more important in determining BMI as the BMI classification increases. For example, the coefficient for the PA variable among those who are in the obese class I BMI classification is -0.0010 for each minute per week of moderate PA. This means that all things being equal, as more minutes per week of moderate PA are performed, BMI is reduced by the coefficient value. However, for those in the obese class II and III BMI classifications, the coefficient for minutes per week of moderate PA is -0.0020, a 100% increase in the effect of this variable on BMI. Hence, we can use the statistically significant coefficients to begin identifying relationships between variables of PA, sedentary behavior, and BMI according to BMI classification.

**Table 2:** Simultaneous Quantile Regression Results--by Body Mass Index Classification

*Dependent Variable: Body Mass Index*						
*Model Parameters*						
Observations = 1,770						
Iterations = 100						
Categories = 5						
Independent Variables	Coefficient	Bootstrap Standard Error	t	*p*	95% Confidence Interval	
					Lower	Upper
**Normal Weight (n = 477)**						
Age	-0.04	0.01	-3.27	**0.001**	-0.07	-0.02
Sex (1 = Male, 2 = Female)	-1.75	0.45	-3.92	**<0.001**	-2.63	-0.88
Non-Hispanic White	0.29	0.58	0.50	0.614	-0.85	1.43
African-American	0.10	1.07	0.09	0.925	-2.00	2.20
Hispanic	1.51	0.74	2.05	**0.041**	0.06	2.96
Rurality (0 = Urban, 1 = Rural)	0.53	0.45	1.18	0.238	-0.35	1.42
Educational Attainment (1 = First Grade, 17 = Graduate School)	-0.04	0.08	-0.56	0.574	-0.19	0.11
Minutes per week of moderate physical activity	-0.0004	0.0004	-0.83	0.407	-0.0010	0.0005
Hours per day spent watching television	0.35	0.19	1.84	0.066	-0.02	0.71
*Constant*	*26.28*	*2.06*	*12.75*	*<0.001*	*22.24*	*30.33*
**Overweight (n = 664)**						
Age	-0.07	0.01	-4.91	**<0.001**	-0.09	-0.04
Sex (1 = Male, 2 = Female)	-1.32	0.30	-4.37	**<0.001**	-1.91	-0.72
Non-Hispanic White	1.48	0.62	2.39	**0.017**	0.27	2.69
African-American	3.8	1.12	3.38	**0.001**	1.59	6.00
Hispanic	2.15	0.75	2.88	**0.004**	0.68	3.62
Rurality (0 = Urban, 1 = Rural)	0.82	0.34	2.41	**0.016**	0.15	1.48
Educational Attainment (1 = First Grade, 17 = Graduate School)	-0.13	0.07	-1.72	0.086	-0.27	0.02
Minutes per week of moderate physical activity	-0.0006	0.0005	-1.29	0.197	-0.0020	0.0003
Hours per day spent watching television	0.87	0.15	5.78	**<0.001**	0.57	1.16
*Constant*	*31.45*	*1.66*	*18.96*	*<0.001*	*28.20*	*34.7*
**Obese Class I (n = 427)**						
Age	-0.15	0.01	-10.33	**<0.001**	-0.18	-0.12
Sex (1 = Male, 2 = Female)	-0.63	0.39	-1.61	0.107	-1.39	0.14
Non-Hispanic White	2.14	0.94	2.27	**0.023**	0.29	3.98
African-American	5.61	1.48	3.80	**<0.001**	2.71	8.51
Hispanic	3.11	1.33	2.34	**0.020**	0.50	5.72
Rurality (0 = Urban, 1 = Rural)	0.49	0.38	1.29	0.197	-0.26	1.24
Educational Attainment (1 = First Grade, 17 = Graduate School)	-0.36	0.10	-3.68	**<0.001**	-0.56	-0.17
Minutes per week of moderate physical activity	-0.0010	0.0004	-2.77	**0.006**	-0.0020	-0.0003
Hours per day spent watching television	1.02	0.19	5.36	**<0.001**	0.65	1.40
*Constant*	*42.15*	*2.15*	*19.62*	*<0.001*	*37.93*	*46.36*
**Obese Class II (n = 177)**						
Age	-0.19	0.03	-6.46	**<0.001**	-0.25	-0.13
Sex (1 = Male, 2 = Female)	0.44	0.59	0.74	0.462	-0.73	1.60
Non-Hispanic White	2.63	1.58	1.67	0.095	-0.46	5.72
African-American	8.25	2.21	3.74	**<0.001**	3.92	12.58
Hispanic	2.50	1.32	1.90	0.058	-0.08	5.08
Rurality (0 = Urban, 1 = Rural)	-0.07	0.67	-0.11	0.915	-1.39	1.25
Educational Attainment (1 = First Grade, 17 = Graduate School)	-0.53	0.13	-3.96	**<0.001**	-0.80	-0.27
Minutes per week of moderate physical activity	-0.0020	0.0006	-3.12	**0.002**	-0.0030	-0.0007
Hours per day spent watching television	0.91	0.24	3.81	**<0.001**	0.44	1.37
*Constant*	*49.62*	*3.77*	*13.16*	*<0.001*	*42.22*	*57.01*
**Obese Class III (n = 236)**						
Age	-0.25	0.06	-4.12	**<0.001**	-0.37	-0.13
Sex (1 = Male, 2 = Female)	2.92	1.17	2.50	**0.012**	0.63	5.21
Non-Hispanic White	4.64	2.64	1.76	0.079	-0.53	9.81
African-American	12.14	4.74	2.56	**0.011**	2.85	21.44
Hispanic	1.98	2.39	0.83	0.407	-2.70	6.66
Rurality (0 = Urban, 1 = Rural)	1.36	1.14	1.19	0.233	-0.88	3.60
Educational Attainment (1 = First Grade, 17 = Graduate School)	-0.62	0.26	-2.35	**0.019**	-1.14	-0.10
Minutes per week of moderate physical activity	-0.0020	0.0006	-3.76	**<0.001**	-0.0040	-0.0010
Hours per day spent watching television	1.87	0.45	4.18	**<0.001**	0.99	2.74
*Constant*	*50.91*	*6.48*	*7.85*	*<0.001*	*38.20*	*63.62*

### Simultaneous quantile regression--similarity of effect by BMI classification

The F tests in Figures 1 and 2 evaluate the null hypothesis that the effect of each independent variable on BMI is the same by BMI classification. Rejecting the null hypothesis (i.e., when an F statistic yields a *p*-value less than the alpha level of statistical significance of 0.05) indicates a heterogeneity effect of the independent variable on BMI by BMI classification. For example, when evaluating the effect of watching television on BMI classification (the graph in the upper left quadrant of Figure 1), the F statistic measuring the effect of watching television between normal weight BMI classification and overweight BMI classification is 5.57 which generates a *p*-value of 0.018. This allows us to reject the null hypothesis (since the *p*-value is below the alpha level of significance of 0.05) that the effect of watching television on BMI is the same between normal weight BMI classification and overweight BMI classification. Using these analyses, it becomes clear that variables relating to PA and sedentary behavior become more meaningful among participants in higher BMI classifications.

Figure 1 illustrates the effect of watching television, moderate PA, self-reporting as African-American, and self-reporting as Hispanic on BMI classification. The effect of watching television on BMI was statistically significant for all BMI classifications except normal weight. The difference from normal weight and overweight demonstrated a positive heterogeneous effect. The effect of moderate PA on BMI was statistically significant for all BMI classifications except normal weight and overweight. The difference between obese class I and obese class II demonstrated a negative heterogeneous effect. The effect of self-reporting as African-American on BMI was statistically significant for all BMI classifications except normal weight. The difference between normal weight and overweight demonstrated a positive heterogeneous effect. The effect of self-reporting as Hispanic was only statistically significant for the classifications of normal weight, overweight, and obese class I. None of the differences between BMI classifications were statistically significant.

Figure 2 illustrates the effect of age, sex, education, and rurality on BMI classification. All classifications of age were statistically significant. The difference between overweight and obese class I demonstrated negative heterogeneous effect. The difference between obese class I and obese class II also demonstrated negative heterogeneous effect. The effect of sex on BMI was statistically significant for normal weight, overweight, and obese class III. The difference between obese class II and obese class III demonstrated positive heterogeneous effect. The effect of educational attainment on BMI was statistically significant for all BMI classifications except normal weight and overweight. The difference between normal weight and overweight demonstrated negative heterogeneous effect. The effect of rurality on BMI was only statistically significant for the overweight classification.

## Discussion

### The effects of PA and sedentary behavior on BMI classification are not homogeneous

This study has addressed the concept of sedentary behavior and PA by looking specifically at how watching television and engaging in moderate levels of PA effect BMI classification. The effect of watching television more than doubled between the normal weight classification and the overweight classification. For those in the overweight classification, an extra 2 h spent watching television per day was associated with nearly one additional BMI point. But, this finding was not consistent across all BMI classifications. Watching television was only incrementally detrimental to BMI when viewed from the perspective of moving from normal weight to overweight. Among respondents who were already overweight, a similar effect was not observed. Conversely, the effect of moderate PA on BMI was greater for those in higher BMI classifications than for those in lower BMI classifications. Specifically, the transition from obese class I to obese class II demonstrated a significant decrease in BMI with increased moderate PA (F = 4.54, *p *= 0.033). This finding suggests there is much to be gained from engaging individuals with higher BMI in moderate PA.

Our findings about the heterogeneous effect of PA is somewhat supportive of Cooper and colleagues' [28] finding that adults of differing BMI classifications also had differing levels of PA. Our finding supplements their study by isolating the statistical influence of differing levels of PA on BMI at different BMI classifications.

### Socio-demographic effects on BMI classification are noteworthy

This study demonstrates the significant association of socio-demographics with BMI classification. Self-reporting as African-American had a significant effect on BMI classification which increased as BMI increased. For those in the overweight BMI classification, self-reporting as African-American was associated with nearly four additional BMI points. This effect was nearly four times as great as the effect of self-reporting as African-American in the normal weight BMI classification. Conversely, self-reporting as Hispanic was only significantly associated with lower BMI classifications such as normal weight, overweight, and obese class I. However, the effect of self-reporting as Hispanic was homogenous across these categories.

Age was significantly associated with all BMI classifications. Among higher levels of BMI classification, the effect of increasing age became stronger. For those in the obese class III BMI classification, each additional year aged was associated with a reduction of one quarter of a BMI point. Self-reporting as male was also significantly associated with lower BMI among the two lowest BMI classifications, normal weight and overweight. However, among the highest BMI classification, obese class III, self-reporting as female was significantly associated with higher BMI. The difference in the effect of being female on BMI was also significantly higher for those in the obese class III BMI classification versus the obese class II BMI classification. Self-reporting as female among the obese class III BMI classification was associated with nearly 3 additional BMI points. Educational attainment was also significantly related to BMI among the higher BMI classifications; obese class I, obese class II, and obese class III. For every additional year of education attained among those in the obese class I BMI classification, the effect on BMI was a reduction of a third of a BMI point. Finally, there did not appear to be significant results to report based on participants' residential rurality. It appeared this variable was of less importance than other socio-demographic variables.

### Is it time to exercise?

A noteworthy finding in this study is the effect moderate PA had on BMI among those in the "obese class III" BMI classification, also known as the "morbidly obese". Part of the challenge in addressing PA for the morbidly obese is overcoming barriers endemic to physical conditions of obese individuals. One such barrier is the perception of breathlessness associated with exercise [29]. This barrier can prevent obese individuals from beginning an exercise regimen. However, there is encouraging evidence that training programs incorporating respiratory muscle development can be successful in helping obese individuals reach their PA goals, improve their metabolic health, and sustain their level of exercise [29]. In addition, mobility limitations of obese individuals caused by diseases such as osteoarthritis and joint pain, must be taken into account when planning appropriate PA programs [30]. Another barrier to PA is motivation, particularly because obese individuals often report a lack of energy or feeling too tired to exercise [31]. These are all unique barriers to PA that programs must recognize in order to be successful.

### Targeted interventions are needed

In addition to indicating the heterogeneous effect of sedentary behaviors among BMI classifications, our study also highlights the role of socio-demographic factors among the various degrees of obesity. While the coefficients in our study identified the unique effect of each independent variable on BMI, the results indicate future research could benefit by examining interaction effects of sedentary and lifestyle behaviors with select demographic groups such as Hispanics and African-Americans. Such analysis could be a powerful complement to the broader findings of our study, and likely highlight the need to tailor interventions not only toward individual BMI classifications, but also toward the multi-cultural composition of those classifications. Addressing the unique needs of the Hispanic [32] and African-American populations [33] can improve the chances of developing programs that successfully improve the health of these populations.

### Limitations

Our study used a unique statistical method to analyze the homogeneity of effect between sedentary behaviors, socio-demographics, and BMI classification. However, simultaneous quantile regression does not have a true equivalent of the coefficient of determination which makes it difficult to establish how much of the variance in the dependent variable was explained with the model. Further, while chronic diseases were recognized in the selection criteria, individual chronic disease characteristics were not analyzed in this study. Future studies would benefit from incorporating individual disease variables as well as individual values of lipoprotein lipase (LPL), low-density lipoprotein cholesterol (LDL-C), blood sugar, and other biometric markers where appropriate. Finally, the data analyzed in this study was self-reported from several communities in central Texas. As a result, it has limited generalizability. It must also be noted the BVHA solicited households for individual responses to the study instrument. As such, individuals not living in households are likely not well represented in the survey results.

## Conclusion

This study found the association of PA and sedentary behavior with BMI is not homogenous across BMI classifications. In fact, PA and sedentary behavior appear to become more important to those who are obese to morbidly obese. This indicates public health efforts must differentiate interventions for individuals of different BMI classifications. While the association between these variables and BMI is well accepted in the literature, this study identifies the degree to which these aspects of life matter to BMI management among those of varying BMI classifications.

## Competing interests

The authors declare that they have no competing interests.

## Authors' contributions

JD conceived of the study and conducted the background research, descriptive statistics, and the simultaneous quantile regression modeling. MS refined the statistical analyses and drafted several portions of the manuscript with specific focus on the methods section. MB framed the obesity concepts and assisted in editing and drafting the content specific portions of the manuscript with specific focus on the discussion section. MO assisted in drafting the abstract, research question, conclusion, and performed extensive editing of the manuscript. All authors read and approved the final manuscript.

## Pre-publication history

The pre-publication history for this paper can be accessed here:

http://www.biomedcentral.com/1471-2458/11/926/prepub

## References

[B1] Ford ES Li C Zhao G Tsai J Trends in obesity and abdominal obesity among adults in the United States from 1999-2008 Int J Obes 2011 35 5 736 743 10.1038/ijo.2010.186 20820173

[B2] WHO http://apps.who.int/bmi/index.jsp?introPage=intro_3.html

[B3] Poirier P Giles TD Bray GA Hong Y Stern JS Pi-Sunyer FX Eckel RH Obesity and cardiovascular disease: pathophysiology, evaluation, and effect of weight loss: an update of the 1997 American Heart Association Scientific Statement on Obesity and heart disease from the Obesity Committee of the Council on Nutrition, Physical activity, and Metabolism Circulation 2006 113 6 898 918 10.1161/CIRCULATIONAHA.106.171016 16380542

[B4] Abdullah A Wolfe R Stoelwinder JU de Courten M Stevenson C Walls HL Peeters A The number of years lived with obesity and the risk of all-cause and cause-specific mortality Intl J Epidemiol in press 10.1093/ije/dyr018 21357186

[B5] Hammond RA Levine R The economic impact of obesity in the United States Diabetes Metab Syndr Obes 2010 3 285 295 21437097 10.2147/DMSOTT.S7384 PMC3047996

[B6] Sturm R Increases in morbid obesity in the USA: 2000-2005 Public Health 2007 121 7 492 496 10.1016/j.puhe.2007.01.006 17399752 PMC2864630

[B7] Deal EN Hollands JM Reichley RM Micek ST Characteristics of patients with morbid obesity at an academic medical center Am J Health Syst Pharm 2010 67 19 1589 1590 10.2146/ajhp100086 20852156

[B8] Mondolfi RN Jones TM Hyre AD Raggi P Muntner P Comparison of percent of United States adults weighing > or = 300 pounds (136 kilograms) in three time periods and comparison of five atherosclerotic risk factors for those weighing > or = 300 pounds to those < 300 pounds Am J Cardiol 2007 100 11 1651 1653 10.1016/j.amjcard.2007.06.072 18036363

[B9] Arterburn DE Maciejewski ML Tsevat J Impact of morbid obesity on medical expenditures in adults Int J Obes 2005 29 3 334 339 10.1038/sj.ijo.0802896 15685247

[B10] Owen N Sparling PB Healy GN Dunstan DW Matthews CE Sedentary behavior: emerging evidence for a new health risk Mayo Clin Proc 2010 85 12 1138 1141 10.4065/mcp.2010.0444 21123641 PMC2996155

[B11] Owen N Healy GN Matthews CE Dunstan DW Too much sitting: the population health science of sedentary behavior Exerc Sport Sci Rev 2010 38 3 105 113 10.1097/JES.0b013e3181e373a2 20577058 PMC3404815

[B12] Katzmarzyk PT Physical activity, sedentary behavior, and health: paradigm paralysis or paradigm shift? Diabetes 2010 59 11 2717 2725 10.2337/db10-0822 20980470 PMC2963526

[B13] Proper KI Singh AS van Mechelen W Chinapaw MJM Sedentary behaviors and health outcomes among adults: a systematic review of prospective studies Am J Prev Med 2011 40 2 174 182 10.1016/j.amepre.2010.10.015 21238866

[B14] Blass EM Anderson DR Kirkorian HL Pempek TA Price I Koleini MF On the road to obesity: television viewing increases intake of high-density foods Physiol Behav 2006 88 4 597 604 10.1016/j.physbeh.2006.05.035 16822530

[B15] Wijndaele K Brage S Besson H Khaw K Sharp SJ Luben R Wareham NJ Ekelund U Television viewing time independently predicts all-cause and cardiovascular mortality: the EPIC Norfolk study Int J Epidemiol 2011 40 1 150 159 10.1093/ije/dyq105 20576628

[B16] Centers for disease control and prevention http://www.cdc.gov/physicalactivity/professionals/reports/index.html

[B17] Church T Exercise in obesity, metabolic syndrome, and diabetes Prog Cardiovasc Dis 2011 53 6 412 418 10.1016/j.pcad.2011.03.013 21545927

[B18] Smith DW Griffin Q Fitzpatrick J Exercise and exercise intentions among obese and overweight individuals J Am Acad Nurse Pract 2011 23 2 92 100 10.1111/j.1745-7599.2010.00582.x 21281375

[B19] Center for Community Health Development Texas A&M Health Science Center School of Rural Public Health Brazos Valley Health Assessment Executive Report 2010 (9/25/2010).

[B20] Centers for disease control and prevention http://www.cdc.gov/nchs/nhanes.htm

[B21] Centers for disease control and prevention http://www.cdc.gov/brfss/index.htm

[B22] Centers for disease control and prevention http://www.cdc.gov/obesity/defining.html

[B23] Wang Y Beydoun MA The obesity epidemic in the United States--gender, age, socioeconomic, racial/ethnic, and geographic characteristics: a systematic review and meta-regression analysis Epidemiol Rev 2007 29 6 28 10.1093/epirev/mxm007 17510091

[B24] Yousefian A Leighton A Fox K Hartley D Understanding the rural food environment--perspectives of low-income parents Rural Remote Health 2011 11 2 1631 1631 21513422

[B25] Economic Research Service http://www.ers.usda.gov/Data/UrbanInfluenceCodes/

[B26] Fousekis P Lazaridis P The demand for selected nutrients by Greek households: an empirical analysis with quantile regressions Agric Econ 2005 32 3 267 279 10.1111/j.1574-0862.2005.00325.x

[B27] Variyam JN Blaylock J Smallwood D Characterizing the distribution of macronutrient intake among U.S. adults: a quantile regression approach Am J Agric Econ 2002 84 2 454 466 10.1111/1467-8276.00310

[B28] Cooper AR Page A Fox KR Misson J Physical activity patterns in normal, overweight and obese individuals using minute-by-minute accelerometry Eur J Clin Nutr 2000 54 12 887 894 10.1038/sj.ejcn.1601116 11114687

[B29] Frank I Briggs R Spengler CM Respiratory muscles, exercise performance, and health in overweight and obese subjects Med Sci Sports Exerc 2011 43 4 714 727 10.1249/MSS.0b013e3181f81ca2 20798653

[B30] Bliddal H Christensen R The management of osteoarthritis in the obese patient: practical considerations and guidelines for therapy Obes Rev 2006 7 4 323 331 10.1111/j.1467-789X.2006.00252.x 17038126

[B31] Cannioto RA physical activity barriers, behaviors, and beliefs of overweight and obese working women: a preliminary analysis WSPAJ 2010 19 1 70 85

[B32] Kohlbry P Nies MA Hispanic women and physical activity: a community approach Home Health Care Manage Prac 2010 22 2 89 95 10.1177/1084822309331576

[B33] Rimmer JH Hsieh K Graham BC Gerber BS Gray-Stanley J Barrier removal in increasing physical activity levels in obese African American women with disabilities J Womens Health 2010 19 10 1869 1876 10.1089/jwh.2010.1941 20815739

